# How Did Corona Crisis Managers in Germany Make Sense of the Psychosocial Situation?

**DOI:** 10.1007/s41125-022-00086-8

**Published:** 2022-10-03

**Authors:** Nils Lüttschwager, Daniela Stelzmann, Lars Gerhold, Sebastian Sterl

**Affiliations:** Institute of Computer Science (Department of Mathematics and Computer Science), Freie Universität Berlin, Carl-Heinrich-Becker-Weg 6-10, 12165 Berlin, Germany

**Keywords:** Corona crisis, Crisis management, Psychosocial information, Sensemaking, Situation picture

## Abstract

Alongside its immediate consequences for physical health, the current Coronavirus pandemic and the associated containment measures have led to multiple psychosocial consequences for the population. While virus containment is the main motive of crisis management, there is, so far, little evidence on how crisis management actors consider findings about the psychosocial state of the German population. This paper therefore examines the role of psychosocial consequences within the work of crisis management organizations during the Corona pandemic in Germany. Against the theoretical background of the sensemaking concept, 14 qualitative interviews were conducted with decision-makers from municipal and state administrations, public health departments, aid organizations, and critical infrastructure organizations. Our results indicate that crisis managers perceive aspects related to the psychosocial situation as relevant, but in a very selective way. They use different and often non-scientific sources to acquire knowledge about the psychosocial consequences. In sum, these aspects do not play a major role in decision-making processes. We argue that the perception and processing of psychosocial consequences depend in particular on the organizational context such as goals, plausibility assumptions, identity conceptions, and problem frames. In order to extend theoretical models of psychosocial crisis management, more detailed knowledge of sensemaking processes in crisis management organizations is necessary.

## Introduction

The emergence of the current Coronavirus pandemic poses a threat not only to people’s physical health, but also acts as a stressor affecting their mental well-being (Daly and Robinson 2022). Since the beginning of the pandemic, a sustainable number of studies has documented the manifold and mostly negative psychosocial consequences, which we generally understand as the result of individual and social processes of experiencing and dealing with crises or disasters.^1^ These consequences can be caused by the Coronavirus itself or by infection control measures (e.g., social distancing measures, school closures). In Germany, studies indicate negative effects of social distancing measures on mental health and well-being in various population groups (Ravens-Sieberer et al. 2021; Benke et al. 2020), the increase in various forms of violence (Clemens et al. 2021; Jung et al. 2020), or the increase in stress among parents (e.g., Buecker et al. 2020; Zinn and Bayer 2021).

Psychosocial consequences represent another challenge for decision-makers in governments and public administrations, who find themselves confronted with a set of new and complex challenges. Especially in the beginning of the pandemic, crucial decisions had to be made on an uncertain information basis, while consequences were difficult to assess. While containing the virus is a central goal of crisis management, it soon became evident that other effects of the pandemic policy, such as economic collapses, threats to educational achievement, or psychosocial consequences, also posed additional challenges to crisis management (Boin et al. 2021). Hence, decisions in crisis management were directly or indirectly linked to people’s psychosocial well-being (see also Dückers et al. 2017).

In general, knowledge about psychosocial consequences can, for example, provide political authorities or public health organizations with an information basis to enable them to provide psychosocial support more effectively to those affected (Beerlage 2021). On the other hand, insights into the experience and handling of the population can also potentially improve the planning, implementation, or evaluation of crisis management measures (Osborne et al. 2021), e.g., with regard to questions of adequate crisis and risk communication (Boin 2015) or the consideration of the specific needs of individual population groups, which can change in different phases after a crisis or disaster (Rao 2006).

However, the extent to which psychosocial aspects play a role in the work of Corona crisis management in Germany remains unclear. Research in political science and public administration focuses particularly on the structure (Hegele and Schnabel 2021), challenges (Blum et al. 2021; Franzke 2020), measures (Behnke and Person 2022; Blum and Kuhlmann 2021; Dostal 2020), or performance (Behnke 2020) of federal-state Corona crisis management. Local governments as well as crisis management teams are studied from a psychological and administrative science perspective regarding existing challenges, resources, and effective behavioral strategies (Thielsch et al. 2020; Eckhard et al. 2021). An initial study by Kuhlmann et al. (2021) indicates that data from social science studies, for example on the social and economic consequences of containment measures, were hardly considered in pandemic management decisions. At the same time, experts from the field of psychosocial emergency care and social science disciplines have complained about a lack of consideration of findings regarding the psychosocial situation in the work of Corona crisis teams in Germany (e.g., Hering et al. 2021; Zitelmann et al. 2020).

In our study, we therefore address the question of the extent to which information about psychosocial consequences plays a role in the work of Corona crisis managers in Germany. Based on 14 interviews with experts from Corona crisis management in Germany, we investigate to what extent crisis management actors perceive aspects regarding the psychosocial situation as relevant, through which sources they acquire knowledge, and what role these aspects play in decision-making. We argue for framing these questions as a sensemaking problem in order to comprehensively explain processes of perception, evaluation, and decision-making. After presenting our theoretical framework and methodological approach, we lay out the results of our qualitative content analysis. Finally, we discuss the results and provide directions for further research.

## Theoretical Framework and Research Questions: Pandemic Crisis Management in a Sensemaking Perspective

The Coronavirus pandemic can without doubt be described as “[a]n unplanned, unusual, unexpected, incalculable, and untested situation that places special demands on those acting” (Jäger et al. 2016, p. 3) and is therefore highly relevant for crisis management worldwide. Following common definitions in the field of civil protection (BBK 2019) as well as crisis management research (e.g., Dückers et al. 2017; Boin 2015; Franzke 2020), crisis management includes all measures that relate to the preparation, detection, management, and follow-up of crises.

In Germany, crisis management in general is based on the federal structure of the state, which is divided politically between the federal government and the states and distinguishes between the federal government, the federal states, and the municipalities at the administrative level (Geier 2017). The states and municipalities traditionally have a central function in the area of disaster management (*Katastrophenschutz)*, which mainly comprises the tasks and measures of non-police and non-military security. For instance, the federal states have legislative powers and technical supervision for internal security tasks (see Articles 30 and 70 of the German Constitution). On the other hand, municipalities are not only responsible for carrying out a large number of operational tasks of disaster management but also form the broad basis of German civil protection with the municipally based aid organizations, fire departments, rescue services and companies as well as volunteers (Geier 2017, p. 96ff.; Pohlmann 2015; Terberl 2015).

The states and municipalities also have essential tasks and competencies in Corona crisis management. The Infection Protection Act (*Infektionsschutzgesetz*), as the legal basis for pandemic management, primarily leaves the legal authority to introduce infection protection ordinances to the states. As the central federal authority for disease surveillance and prevention, the Robert Koch-Institute (RKI) is legally responsible, among other things, for monitoring and evaluating the epidemiological situation, deriving recommendations for infection control measures, and providing these information and recommendations to the public and the public health authorities (see RKI 2020, Verwaltungsvorschrift-IfSG-Koordinierung—IfSGKoordinierungs-VwV). The information provided by the RKI thus constitutes a central source of information for pandemic crisis management measures for the organizations and authorities of civil protection and public health services in the states and municipalities. To ensure a joint national approach, the federal and state governments have coordinated their efforts in common regular conferences (*Bund-Länder-Gespräche*). Operational crisis management was carried out by the municipalities, which implemented and monitored legal ordinances and ensured the detection of infections and contact tracing through the regional health offices and transmitted this information to the RKI (Franzke 2020, p. 326ff.; see also Kersten and Rixen, 2021). In addition to the authorities, aid organizations also played an important role during the pandemic, for example by providing protective materials, organizing ambulance transport, or setting up vaccination sites. Critical infrastructure organizations focused on securing supply systems.

While psychosocial consequences of crises and disasters have been the focus of scientific and political discussion for many years (cf. Norris and Elrod 2006; Bonanno et al. 2010; WHO 1992), according to Dückers et al. (2017), there have been few conceptual approaches linking the management of psychosocial consequences with typical crisis management tasks. Dückers et al. (2017) therefore develop a model of psychosocial crisis management (PCM) that allows us to narrow down the object of study. The core tasks of psychosocial crisis management in this model consist of sensemaking, decision-making, coordination, meaning making, account giving, and learning. In our study, we draw attention to the sensemaking process, because it is needed “to assess the event and its potential effects on the exposed population. This assessment should identify psychosocial risks, needs and problems, risk factors [, and] (insufficient) capacity to adapt, preferably guided by lessons learned from earlier situations” (Dückers et al. 2017, p. 100). This means that whether or not psychosocial aspects play a major role in crisis management greatly depends on crisis managers’ sensemaking of the situations they face.

**Fig. 1:**
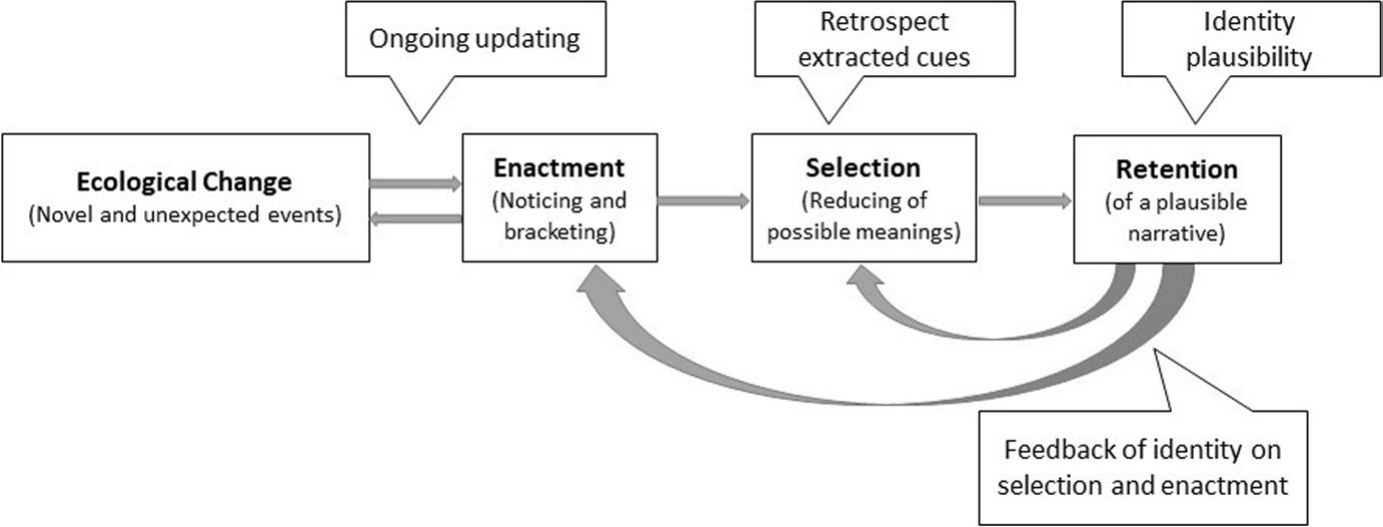
The Sensemaking cycle based on Weick et al. (2005, p. 414)

The concept of sensemaking was developed in particular by Karl Weick (1995) and describes the active construction of social reality which “involves the ongoing retrospective development of plausible images that rationalize what people are doing” (Weick et al. 2005, p. 409).^2^ Processes of sensemaking appear when novel, unexpected or confusing events arise in the stream of experience of individuals and invoke questions like “What is going on?” and “Now what?” (see Maitlis and Christianson 2014, p. 58). Sensemaking therefore “allows people to deal with uncertainty and ambiguity by creating rational accounts of the world that enable action” (Maitlis 2005, p. 21). As a central part of organizing, sensemaking works as a temporally repetitive sequence of activities in which environmental anomalies (ecological change) are perceived by social actors (noticing and bracketing) and, through a combination of “retrospective attention, mental models, and articulation” (Weick et al. 2005, p. 414), the range of possible meanings is reduced (selection) so that eventually a plausible narrative is solidified (retention) (see Fig. 1). Particularly in dynamic and critical contexts, where there is a need to establish a coherent understanding to enable collective action, sensemaking is an important and challenging task (Maitlis 2005, p. 21).

The Corona crisis has undoubtedly created such an uncertain and dynamic environment (Christianson and Barton 2021; Boin et al. 2021) that has led to disruptions and changes in routine processes across most areas of society. In this respect, the Corona crisis is a trigger for sensemaking processes. It is important in the context of the long-lasting and dynamic Corona crisis that sensemaking processes do not stop when a crisis is recognized and labeled as such (Boin et al. 2021, p. 21; see also Mills et al. 2010). Sensemaking remains necessary throughout the existence of a crisis, “directing policymakers’ attention to selected cues, propelling them toward some but not other interpretations of how the crisis is developing, guiding them toward some but not other courses of action” (Boin et al. 2021, p. 21). In the Coronavirus pandemic, sense had to be made repeatedly over a long period of time on the basis of a large amount of information about a constantly changing situation (Christianson and Barton 2021).

Against this background, we draw attention to information about the psychosocial consequences. A psychosocial situation picture should include “all information on the (potential) behavior of those directly and indirectly affected as well as their psychosocial needs and assistance requirements” (Helmerichs et al. 2017, p. 294, see also Wahl and Gerhold 2021). Crisis managers can obtain this information in different ways: Informal ways include personal conversations with affected individuals, while more formal ways include investigations or professional diagnoses and the collection of data by conducting surveys (Karutz and Tinla 2021). At the level of a larger community or a country, Dückers et al. (2017) suggest the creation of a health or needs assessment or health monitor, which indicates physical or mental health status (p. 101). In addition, social media channels, press articles or television programs are also possible sources for analyzing emotions and other psychosocial aspects (Dückers et al. 2017).

The aim of this study is to gain a comprehensive understanding of the role of such information in the process of sensemaking on the psychosocial consequences of the Corona crisis in German crisis management. The study seeks to address the following research questions:

- **RQ 1:** To what extent has information on the psychosocial situation been considered relevant for sensemaking processes in Corona crisis management?
- **RQ 2:** Which information channels do crisis managers use to make sense of the psychosocial situation?
- **RQ 3:** Which factors influence the consideration of psychosocial information on decision-making processes in Corona crisis management?

## Methodology

The work of corona crisis management actors in Germany is a relatively unexplored field of research. The expert interviews conducted in this research therefore aimed to explore crisis management actors’ “process knowledge” which includes “insights into courses of action, interactions, organizational constellations, events, etc. in which the interviewees are or were involved” (Bogner et al. 2014, p. 18).

### Participants

Following Bogner et al. (2014), we view experts as those “who, based on specific practical or experiential knowledge that relates to a clearly definable problem area, have created the possibility of structuring the concrete field of action in a meaningful and action-guiding way for others with their interpretations” (p. 13). Experts in this sense occupy certain social positions whereby their opinions, interpretations, and expertise have consequences for other people. Thus, for example, staff members in crisis teams who contribute information or interpretations in decision-making processes are regarded as experts in the above sense.

As a key criterion for inclusion in this study, participants needed to be currently participating or to have previously participated as part of a crisis management team in the Corona crisis. Furthermore, the aim was to recruit crisis management team members from organizations with different crisis management tasks and decision-making levels. The selection of experts in this study was not based on criteria of statistical representativeness. Rather, the aim was to draw a so-called purposive sample (Flick 2019) that represents different crisis management actors in organizations involved in Corona crisis management. Generalizations in the sense of identifying repeating social mechanisms for the subject area are thus acceptable (Przyborski and Wohlrab-Sahr 2021). Table 1 gives an overview of the participants, their institutional backgrounds and job descriptions. Most of the interviewees come from municipal administrations (five out of 15). Of the remaining participants, two work as chief medical officers in health departments, two work as crisis team leaders or coordination team leaders within critical infrastructure organizations, two hold senior positions in a civil protection organization, and two work as crisis team leaders in aid organizations. At the state level, one interviewee works within the crisis team of the Ministry of Health, and the other works as a Coordination Group Leader in the office of a Minister-President. On the one hand, we recruited participants through research project networks and personal job contacts. On the other hand, we researched organizations at the community level, such as municipal governments or health departments, and then requested for an interview by phone or email. The participants work in the federal states of North Rhine-Westphalia (7), Berlin (4), Schleswig-Holstein (2), Mecklenburg-Western Pomerania (1) and Lower Saxony (1). Except for two persons (No. 12, 13), all participants belong to different organizations.

**Table 1:** Institutional context and job description of study participants

No.	Institutional context	Job description
1	State Administration (Minister-President’s Office)	Coordination Group Leader
2	State Administration (Ministry of Health)	Crisis Management Team Member
3	Aid Organization	Crisis Management Team Leader
4	Aid Organization	Crisis Management Team Leader
5	Critical Infrastructure	Crisis Management Team Leader
6	Critical Infrastructure	Head of Coordination Team
7	Public Health Department	Chief Medical Officer
8	Public Health Department	Chief Medical Officer
9	Civil Protection Organization	Head of Management Staff
10	Civil Protection Organization	Deputy Head of Unit
11	Municipal Administration	Crisis Management Team Leader
12	Municipal Administration	Deputy Crisis Management Team Leader
13	Municipal Administration	Crisis Management Team Member/School Sector
14	Municipal Administration	Mayor
15	Municipal Administration	Crisis Management Team Member/Press Office

### Materials and Procedure

We used the same interview guideline for all interviews and, except for one, all interviews were carried out by the first author via videoconferencing software (Cisco WebEx Meetings). The interview guide was divided into four thematic blocks: 1. professional background of the crisis management actor, 2. crisis management tasks and work routines, 3. perception and handling of information on the psychosocial situation, and 4. requirements for the use of information on the psychosocial situation in crisis management. The participants gave their full consent to be subjects of this study. All information that could indicate the identity of the interviewees was anonymized. The interviews were conducted in German and recorded, and took, on average, about one hour. All the interviews were transcribed and formed the basis for the qualitative data analysis.

### Data Analysis

We analyzed the transcripts in a multi-stage procedure according to the method of structured content analysis following Mayring (2015) and Kuckartz (2018), and using the software MAXQDA 2020. In an iterative process of inductive-deductive analysis, we then developed a system of categories and related coded segments in the interview data (Kuckartz 2018) which help in addressing the research questions. In the first step of the analysis, the first author read the entire interview several times. In the second step, the first author coded statements by means of a deductive category system developed on the basis of the PCM model proposed by Dückers et al. (2017). In order to be added to a single category, the interview segments must match the given definitions and criteria of the category. In the case of a text passage not fitting into the existing category system, either the category system was expanded, diversified (e.g., into subcategories) or adjustments were made to the definitions or criteria of individual categories. In the last step, the first author presented and discussed the identified categories with the co-authors. If disagreements arose, the coded text segments were revisited and their meaning discussed until a consensus was reached within the research group.

The category system constitutes the central outcome of the study, as this is continually reviewed and refined through the ongoing interplay between theory and data analysis (Kuckartz 2018). The category system is divided into three sections. According to Dückers et al. (2017), sensemaking in the PCM model involves assessing “psychosocial risks, needs and problems, risk factors, [and] insufficient capacity to adapt” (p. 100), so these aspects formed the starting point for the first section of the category system “Relevant psychosocial dimensions.” After coding the interviews on the basis of these categories, we decided to develop new main categories, which are part of the final category system. This restructuring of the main categories arose from the insight that the psychosocial aspects identified become relevant in different ways within the organizations. These differences are represented in the newly formulated main categories. The categories of the second section “Used information channels” include the information channels used by crisis managers to assess the psychosocial situation and are also derived from the PCM model by Dückers et al. (2017). The third section “Factors for consideration of psychosocial information on decision-making processes” includes categories that serve as explanatory variables for the missing consideration of psychosocial information and were created inductively from the data.^3^

**Table 2:** Final category system

(1) Relevant psychosocial dimensions (Dückers et al. 2017)	
Integral part of the situation assessment Public opinion/public interest Patients’ well-being Psychosocial stress for organizational members Fear of own infection and transmission to others Dealing with relatives of deceased Increased workload Disruption of work processes Protective measures preventing work execution Interruption of contact with patients Risks for achievement of crisis management goals Public unrest Lack of commitment from volunteers	
(2) Used information channels (Dückers et al. 2017)	
Everyday experiences Organizational expert networks Inquiries from citizens or employees and contact with patients Media coverage Social media Surveys/studies	
(3) Factors for consideration of psychosocial information on decision-making processes	
Underestimation of social science data compared to epidemiological data Priority of infection control Political and public discourse	

For the following presentation of the results of the structuring content analysis, we have formulated summarizing statements and illustrate these with quotes “that best represent a category, so-called ‘anchor examples’” (Gläser-Zikuda 2013, p. 143; authors’ translation).

## Results

### To What Extent Has Information on the Psychosocial Situation Been Considered Relevant for Sensemaking Processes in Corona Crisis Management? (RQ 1)

The extent to which changes in the psychosocial situation are interpreted as problematic, irritating or disruptive for the performance of the Corona crisis management tasks varies. Based on the analysis, we identified four perceptions of psychosocial aspects: *as an integral part of the situation assessment*, *psychosocial stress for organizational members*, *disruptions of work processes*, and *risks for the achievement of crisis management goals*.

*Integral part of the situation assessment* In particular, participants from municipal administrations as well as health authorities see themselves as responsible for monitoring the public mood or the mental well-being of their patients. In accordance with this self-image, decisions within the context of infection control are evaluated with regard to possible negative psychosocial consequences. Community representatives usually subsume the term "psychosocial situation" to include public opinion, acceptance of and compliance with Corona rules, or the perceived needs of the population. They emphasize that psychosocial effects of measures are consistently perceived and "taken into account"—i.e., they were a naturally perceived part of the overall tasks—without this being made explicit. For the head of a health department, psychosocial aspects also belong to an overall assessment of the patient's situation. The psychosocial well-being of quarantined persons is thus always a relevant factor in decisions about further quarantine measures:

And of course the question ‘How are the people?’ is very, very important. But you don't have to talk about that in detail. That is obvious. (Chief Medical Officer/Public Health Department)

*Psychosocial stress for organizational members* For some of the participants, psychosocial consequences of the pandemic become problematic if they affect the routinized functioning of organizational processes, for example, when the mental state of employees is worsening. In this context, the participants report on various kinds of stress that had affected employees of their own organization, especially employees of aid organizations and health offices. These sometimes include employees' fear of their own infection, the fear of infecting family members or high-risk patients, and a general increase in workload. For health department staff responsible for contact tracing, dealing with the relatives of the recently deceased can be an additional burden.

*Disruptions of work processes* Impairments of routinized organizational processes that can lead to negative psychosocial consequences are also considered problematic. On the one hand, this is reflected when employees are no longer able to perform their work due to protective measures. On the other hand, the consequences of infection control measures lead to the fact that the care of patients in the health department, for example, in the area of child, adolescent and adult psychiatry, is limited or completely absent over a longer period of time. These "dried up channels of information" raise concerns about possible deterioration in the mental health of various patient populations.

*Risks for the achievement of crisis management goals* In addition, the participants assess psychosocial consequences for crisis management as relevant if the achievement of the organizational crisis management goals is put at risk by these consequences. For the interviewed representative of the critical infrastructure organization, the psychosocial situation of the population plays a minor role in his or her work, since ensuring the ongoing functioning of companies and providers of critical infrastructure (e.g., supermarkets, water supply, waste disposal) is primarily regarded as a problem that is independent of the psychosocial situation of the population. Only when the population reacts with fear and panic to the pandemic situation the goals of critical infrastructure organizations will be at risk. The situation is similar for the management of the civil protection organization. Negative psychosocial consequences currently play no role within crisis management but the lack of commitment from volunteers is seen as a potential negative psychosocial aspect which could affect the organizational crisis management effort.

### Which Information Channels Do Crisis Managers Use to Make Sense of the Psychosocial Situation? (RQ 2)

The crisis managers interviewed use diverse communication channels to gain insights into the psychosocial situation, rather than one central source. We distinguish the identified information channels according to the extent to which the information collected came directly from the affected persons or was passed on in certain formats, e.g., through media reports (see also Dückers 2017, p. 100f.). The following description therefore begins with more direct processes of information gathering and ends with more indirect processes. A total of six information channels were identified in the interviews: *Everyday experiences*, *organizational expert networks*, *inquiries from citizens or employees and contact with patients*, *media coverage*, *social media* and *surveys/studies*.^4^

*Everyday experiences* The municipal crisis managers use everyday experiences and conversations as a basis for classifying aspects of the psychosocial situation. These experiences include encounters and conversations from the private sphere, informal conversations with co-workers, or unspecified "impressions" from everyday life that are considered relevant knowledge for assessing the public mood. At the same time, the crisis team members' own exposure to the Corona pandemic is cited as a reason to be able to understand the psychosocial situation of citizens:

And one must not forget that all employees who are also on the staff are also affected to a certain extent. […] The whole picture of society is then also reflected in the people on the crisis team. (Deputy Crisis Management Team Leader/Municipal Administration)

*Organizational expert networks* Participants suggest that experts from the organizational environment generate information about the psychosocial situation. Experts from their own organization hand over information because the participants themselves do not have the time resources to evaluate scientific studies. In municipal crisis management, it was the department heads within the municipality who provide the "empirical findings from the municipal institutions" (crisis management team (school sector)/municipal administration), which formed the basis for the assessment of (psychosocial) needs and requirements of specific groups, especially the youth.

*Contact with affected people (citizens, employees, patients)* The requests mentioned by the participants are directed to the responsible persons of the municipal crisis teams as well as to the crisis management of the health offices and include the wish for information or concrete political decisions, such as the (early) opening of local stores. Especially in municipal crisis teams, requests from citizens are used as an information channel assessment of the psychosocial situation. Within aid organizations, psychosocial emergency hotlines for the organization's emergency workers are gaining in importance during crisis management. However, demand from the aid organization's own employees varies: While one aid organization employee interviewed was surprised by "how little it [the hotline] was used by their own employees," the second aid organization employee interviewed pointed to the increased need for psychosocial support for employees who themselves developed fears of infection or requested support needs for their subordinates. Telephone communication with patients conducted as part of contact tracing also plays an important role in the health offices to assess the psychosocial situation. Telephone communication was also used to monitor the psychosocial well-being of patients who had already been treated before the Corona pandemic and for whom direct patient contact was not possible due to the closure of daycare centers and schools.

*Media coverage* Media reports are of little relevance to very few respondents in forming a psychosocial picture of the situation. Different purposes of an evaluation of media products, which include newspaper reports and news, were stated. The respondent from the civil protection organization describes monitoring press coverage as a way to identify areas for action in crisis management early on, enabling rapid organizational action (e.g., early procurement of protective materials), as well as assessing public perception of their organization. The officer from the critical infrastructure sector uses press monitoring as an instrument for identifying regional and supraregional problem situations (e.g., possible supply gaps in the area of food supply).

*Social media* Social media platforms such as Facebook or Twitter play a role in the assessment of the psychosocial situation of the population for the representative of the disaster management organization, the relief organization and two representatives of the municipal crisis management. For the disaster management organization, the posts on social media provide an opportunity to assess the public perception of the civil protection organization. This also makes it possible to assess the extent to which media coverage may have a negative impact on the readiness of the organization's volunteer members. In addition to press inquiries, municipal crisis teams perceive a large number of citizen inquiries via social media, from which they obtain information about the psychosocial situation. In addition to dealing with questions or fears derived from social media postings, misinformation or rumors play a role, to which municipal crisis managers respond with corrections via their own communication channels. Municipal crisis managers further use social media channels to gauge public opinion about municipal Corona policies in general. For example, one mayor interviewed considered comments on his or her Facebook page as "clues" to gauge public sentiment and attitudes about Corona policy.

*Surveys/studies* Scientific studies on the psychosocial situation of the population played a minor role for the crisis management actors interviewed. This was mostly due to a lack of time and personnel resources. An in-depth examination of studies on the psychosocial situation was difficult, for example, for the representative of the aid organization due to the variety of available scientific findings. The mayor interviewed takes population surveys on Corona policy to assess citizen satisfaction with his or her crisis management.

### Which Factors Influence the Consideration of Psychosocial Information on Decision-Making Processes in Corona Crisis Management? (RQ 3)

The majority of the participants state that psychosocial aspects are relevant for their work in crisis management. However, the participants only make very general references to the importance of considering psychosocial aspects. In a few cases, they explicitly explain to what extent these psychosocial information aspects play a decisive role in decision-making. In addition to structural factors such as lack of time, lack of human resources, and a multitude of tasks, the *underestimation of social science data compared to epidemiological data*, *the priority of infection control* and *political and public discourse* are also factors influencing the consideration of psychosocial factors on crisis management decisions.

*Underestimation of social science data compared to epidemiological data* The member of a crisis team within a health authority at the state level describes that those responsible were indeed aware of the psychological stress on children and adolescents caused by school closures. However, these findings do not play any role in concrete decision-making processes—such as the question of school closures and openings—until the epidemiological situation eases and additional scientific findings in the form of studies clarify the negative psychosocial consequences for children and adolescents as a result of school closures. At the same time, "hard" epidemiological indicators are considered to be more informative than "soft" social science study data on psychosocial consequences:

But that's also often difficult, because that's soft data, as everybody knows that the children are suffering, but the corresponding studies are, yes, the data are somehow softer than these very clear [virus] transmission data. So, you know, one person infects 2.5 others under these conditions. That's mathematics and you can then calculate something like that and then pretend that that's the truth. (State Administration (Ministry of Health)/Crisis Management Team Member)

*Priority of infection control* On the other hand, the participants perceive information on the psychosocial situation and are aware of psychosocial consequences, but orient their actions to the "scientific opinion" (Municipal Administration/Mayor) or the goal of "infection control" (Municipal Administration/Deputy Crisis Management Team Leader). Finally, the participants regard infection control as the prevention of deaths. Psychosocial consequences are regarded as a necessary evil secondary to the goal of protecting human life through infection control measures.

*Political and public discourse* The extent to which crisis managers integrate the psychosocial situation as a legitimate argument for or against a particular strategic direction in Corona crisis management also depends on the pandemic situation and the state of the public debate. The member of a state-level health administration crisis team, who also co-authored expert recommendations for the state government, points out that the favorable epidemiological situation in the summer of 2020, with few Corona case numbers, allowed this person to use scientific evidence from studies on negative psychosocial consequences of school closures for children as a policy argument for greater withdrawal of infection control measures. In this case, this participant uses scientific findings on the psychosocial situation strategically as a supporting political argument.

## Discussion: Psychosocial Crisis Management as a Sensemaking Problem

In sum, the results show that standardized procedures for surveying and assessing the psychosocial situation in crisis management organizations are not in place with regard to the current Coronavirus pandemic. Corona crisis management organizations consider very different information on the psychosocial situation as relevant, relying on different practices to collect it and only partially incorporating it into their decisions. In the context of sensemaking theory, we argue that *organizational goals*, *plausibility*, *identity conceptions*, and *problem frames* are central interdependent factors underlying how psychosocial information in different Corona crisis management organizations is perceived, evaluated, and incorporated into decisions.

The extent to which crisis managers include the psychosocial situation in their sensemaking depends primarily on the specific *goals* and *tasks* of the organization, which vary within and between organizations. In most cases, psychosocial aspects have no priority in specific crisis management practices if normal work processes are not affected by them. They are mainly considered relevant when they affect organizational processes negatively, for instance when employees are affected or work routines disrupted, or if they are seen as potential barriers to successful organizational action (see category system, Sect. 1). In most cases, the task fulfillment of the organizations is only marginally influenced by how people inside or outside the organizations are affected by psychosocial consequences. In other words, because central crisis management goals are often not seriously threatened by the psychosocial consequences of the pandemic, these factors do not require much attention in the general sensemaking processes. For example, the monitoring of psychosocial consequences in the population serves, in the case of municipal administrations as well as in civil protection organizations, to evaluate the probability of success of organizational decisions or decision-making. The municipal administrations for example are particularly tasked with implementing state ordinances on infection control and monitoring compliance with them (see Franzke 2020). The extent to which the population accepted and followed the current Coronavirus-related ordinances is not important for achieving this goal.

When psychosocial information is considered relevant, the assessment of the psychosocial situation is often based on unsystematically collected knowledge, for instance from informal or everyday conversations or assessments by colleagues, which is particularly evident in municipal crisis management. Data on the psychosocial situation that aim at “accuracy” (such as scientific studies) are hardly noticed in crisis management. Reasons such as lack of time, high workload, or issues seen as more important or pressing than psychosocial concerns partly explain this. Nevertheless, the interview data indicate that even seemingly “inaccurate” sources (such as everyday impressions or participants’ own concerns) are sufficient to establish *plausibility*. Thus, in order to take meaningful action in community crisis management, it is not essential to be familiar with the current state of research on psychosocial consequences. It seems more important to acquire a “feeling” for the mood in the local population in order to be able to assess reactions to political decisions. Viewing posts on social media channels, fielding inquiries from the general public, and everyday experiences are highly subjective processes, but many interviewees found these sources sufficient as a plausible basis for evaluating political decisions.

Against the background of sensemaking theory, we assume that individual as well as organizational *identities* influence the extent to which psychosocial aspects are perceived as relevant. The aid organization, for example, has established emergency psychosocial care as an institutionalized area of its organization, which it “has always held [it] in high regard” (Crisis Management Team/Aid Organization). This perception of an organization that can provide psychosocial support forms its members’ identity basis for assessing information about psychosocial consequences as relevant in crisis management sensemaking processes.

As the interview with the crisis team member at the State Ministry of Health shows, the act of problem *framing* psychosocial consequences as a clear and tangible issue plays an important role in decision-making and communication about those decisions. Even if negative psychosocial consequences of containment measures on parts of the populace are known and assessed as relevant within crisis management processes, this does not necessarily lead to decisions which aim at improving the psychosocial situation. Scientific results regarding stress felt by children and adolescents are considered less meaningful (“soft data”) compared to (apparently) clear virus transmission data by the participant. The applied framing is thus based on an idea of the objectivity of quantitative data. In this case this problem framing not only leads to a prioritizing of epidemiological metrics, but also serves as a basis of legitimacy in the communication of recommendations for or against containment measures. At the same time, this interview suggests that there are certain “windows of opportunity” (see Zahariadis 2019) for the inclusion of psychosocial evidence in policy justification. When the epidemiological situation seems innocuous enough and public discourse is based less on this theme, windows of opportunity become available to include studies in, for example, the rationale for opening-up measures.

Despite a comparatively small number of interviews, our findings generally support the argument of Kuhlmann et al. (2021) that social science perspectives on the Covid-19 crisis have been less important for decision-making processes than epidemiological indicators (e.g., the seven-day incidence rate or the R number; p. 45), and data on societal and economic impacts have “hardly played a role” (p. 27) in relevant decisions. Our study can follow up on this research by identifying relevant factors regarding how and why specific information is incorporated into Corona crisis management. This study, compared to that of Kuhlmann et al. (2021), also interviewed representatives from political and government agencies as well as aid organizations, public health departments, and critical infrastructure organizations in their role as crisis management organizations.

However, based on our data, it is not possible to make more precise statements about interaction and communication processes within crisis management work, especially in crisis team meetings. The research literature on sensemaking, however, suggests that, especially in temporary social formations that come together (such as crisis teams), for example, linguistic aspects (Cornelissen et al. 2008; O’Leary and Chia 2007), role structure (Bechky 2006; Bigley and Roberts 2001; Meyerson et al. 1996), and interaction practices (Maitlis and Christianson 2014, p. 94f) are relevant factors to understand sensemaking processes in specific situations with different stakeholders (see, for example, Wolbers and Boersma 2013; Wolbers 2021). Thus, questions about patterns of communicative negotiation of meaning across psychosocial aspects must remain unanswered in this study.

## Outlook and Methodological Limitations

Regarding how psychosocial situation information can be more strongly integrated into crisis management processes in the future, this study can provide initial, albeit very general, indicators. It should become clear that a closer consideration of psychosocial aspects is not only a question of providing more scientific data. If the management of psychosocial consequences is not already at least partly a task of crisis management organizations (as in the case of aid organizations or public health departments), they are only partially perceived as relevant problems within work processes. In this study, we find little evidence that they are an important part of the sensemaking in crisis management teams. In crisis management organizations, structures must be established that are designed to recognize and deal with negative psychosocial developments in the organizational environment (as a problem). The interviews suggest that there are very different understandings about what psychosocial consequences are. From a sensemaking perspective, establishing social contexts in which targeted sensemaking about the psychosocial situation takes place can promote the creation of consistent actions (Maitlis 2005). Consequently, sensemaking processes as well as the specific organizational practices of crisis management organizations must be studied as two sides of the same coin—sensemaking must encompass organizations and vice versa.

Due to limitations of time and funding, we were only able to interview a small number of relevant Corona crisis management actors. Since central strategic decisions of Corona crisis management in Germany are made at the federal and state level, expert interviews with crisis management actors at these political and administrative strata would complement the results set out here. Furthermore, the array of experts interviewed here is limited to people from western, northern and eastern Germany; crisis management actors from the south of Germany were not interviewed. Since the challenges and available resources of pandemic management differed across federal states and municipalities during the course of the pandemic, results may be distorted. Furthermore, our findings are based on the memories of individuals who were reconstructing events experienced several months in the past. Process-produced data such as minutes or audio recordings of crisis team meetings may thus paint a more accurate picture of how meaning was generated with respect to the psychosocial aspects of the pandemic’s management. Nevertheless, we believe that our findings are highly relevant for the management of the ongoing crisis. The transition from a pandemic to an endemic phase will not diminish the importance of the questions raised here. Therefore, we hope that our insights might lead to further research that discusses the relevance of psychosocial impacts for crisis management.

## Appendix

See Tables 2,

**Table 3:** Summary of the results for RQ 1

Category	Definition/summary	Example
*Main category*: Integral part of situation assessment	Psychosocial aspects form an integral part of the situation assessment	–
*Subcategory*: Public opinion/public interest	Municipal representatives subsumed under “psychosocial situation” mostly “public opinion,” acceptance of and compliance with pandemic rules, or perceived needs of the population. Psychosocial effects of measures were consistently perceived and “taken into account”—i.e., they were perceived as an integral part of crisis management tasks—without this being made explicit	“But I think I can separate it [the consideration of psychosocial consequences on the population] so badly. Because, actually, it was always the day-to-day business. The impact and then thinking: What can the city do now? What can politics also do at that point?” (Crisis Management Team Leader/Municipal Administration)
*Subcategory*: Patients’ well-being	The mental well-being of quarantined individuals is always an important factor in health department decisions about further quarantine measures	“Well, yes, it always plays a role. You don’t order anything that is not absolutely necessary. And of course, the question ‘How are the people?’ is very, very important. But then you don’t have to talk about that in detail. That’s obvious.” (Chief Medical Officer/Public Health Department)
Main category: Psychosocial stress for organizations’ members	Psychosocial consequences of the pandemic are relevant or problematic for crisis management organizations if they affect the psychosocial health of employees	–
*Subcategory*: Psychological stress for employees	The interviewees report on various kinds of stress that had affected employees of their own organization, especially employees of aid organizations and health offices. These include increased workloads, dealing with people who have lost loved ones, or the burden of constantly wearing protective equipment	“But in the situation, there have been regions where people have been dying like flies in [care] facilities. And that has really been a phenomenon which has also pushed many, many caregivers, professionals to the limit.” (Crisis Management Team Leader/Aid Organization)
*Subcategory*: Fear of own infection and transmission to others	Employees’ fears relating to the risk of contracting the Coronavirus or infecting other people (e.g., their own family and high-risk patients)	“Naturally, fears have been generated among our colleagues. And we have repeatedly tried to minimize these fears by providing information that is as objective and valid as possible. Of course, you can’t get rid of them [the fears].” (Chief Medical Officer/Public Health Department)
*Subcategory*: Dealing with relatives of the deceased	For temporary staff in health departments who were tasked with contact tracing, dealing with relatives of the recently deceased can be an additional stressor	“It’s no small thing when you’re constantly dealing with people who have recently lost a relative. And we’ve had families that were really dramatic cases, then. You don’t experience that if you normally work in the, I don’t know, in the building department and now also once work in the health department. You don’t come into contact with dead people at all in your other work” (Chief Medical Officer/Public Health Department)
Main category: Disruption of work processes	Disruptions to organizational routines that can lead to negative psychosocial consequences are considered problematic	–
*Subcategory*: Protective measures preventing work execution	The introduction of protective measures results in employees being hindered in the normal performance of their work or being unable to perform it. This can also result in an additional burden for employees	“These [the suspension of first aid courses] are things that put pressure on the mind in that people say, ‘Yes, it’s also my job to train people. And it’s important to learn first aid even in a crisis. These are things that you have to endure.” (Crisis Management Team Leader/Aid Organization)
*Subcategory*: Interruption of contact with patients	Measures of contact reduction lead to impairments in the care of patients in the health department in the area of child, adolescent and adult psychiatry over a longer period of time. These "dried up channels of information" raise concerns about possible deterioration of patients' mental health.	“There were, after all, schools and daycare centers closed at some point. And schools and daycare centers are a very important place for our child protection sector, because we get the information from them when a child is not well. […] That made us extremely worried because we suspected that something was brewing.” (Chief Medical Officer/Public Health Department)
*Main category*: Risks for achievement of crisis management goals	In some participants’ view, negative psychosocial consequences could threaten the future achievement of key organizational crisis management objectives. These consequences are therefore not currently relevant, but they do represent a risk for future organizational actions	*–*
*Subcategory*: Public unrest	The psychosocial situation of the population plays a subordinate role, as this phenomenon does not affect crisis management objectives. Only when the behavior of the population reaches a critical threshold in the future does this become problematic for organizations dealing with critical infrastructure	“Of course, the psychosocial situation of the population becomes relevant when the mood shifts, if I may exaggerate. Yes, when people take to the streets, when people realize they can’t get any more cash. When there’s a bank run, when it’s determined that the supermarkets have to close due to high illness rates and the employees, then the people need a supply.” (Head of Coordination Team/Critical infrastructure organization)
*Subcategory*: Lack of commitment from volunteers	Lack of volunteer engagement is seen as a possible negative psychosocial aspect. This could lead to less volunteer engagement with civil protection organizations and could thus negatively affect the work as a whole	“I would perceive it [the psychosocial situation] only when it exceeds a certain threshold. […]. I’m personally interested in these issues, of course. But I can’t say that it played a role in my decisions in the management team. So here it was essentially a matter of internal acceptance. And being seen from the outside.” (Deputy Head of Unit/Civil Protection Organization)

3,

**Table 4:** Summary of the results for RQ 2

Category	Definition/summary	Example
Everyday experiences	Everyday experiences and conversations are used as a basis for classifying aspects of psychosocial situations. These experiences include encounters and conversations from the private domain, informal conversations with employees, or unspecified “impressions” from everyday life which are considered as relevant knowledge for evaluating the public mood, especially by members of municipal administrations	“[We] are all people, too. And everyone on the staff of course goes shopping, goes into town, meets friends. And they talk about the issues there. And if something comes up there along the lines of: ‘Look, there’s this or that rumor circulating among the neighbors again,’ then we have that—that was partly also carried in there [crisis management team].” (Crisis Management Team Leader/Municipal Administration)
Organizational expert networks	Individual experts or groups of experts in the participants' immediate or wider organizational environment provide information about the psychosocial situation. Due to time restrictions, the amount of information, and lack of access, experts from the organizational environment of the participants report on the psychosocial situation of specific social groups	“[T]here is such a variety of information that actually makes a meta-study necessary in order to get through it. I don’t mean that in a bad or disrespectful way. That’s why I didn’t rely on such [scientific] sources, but on statements from my experts.” (Crisis Management Team Leader/Aid Organization)
Contact with affected people (citizens, employees, patients)	Contact with affected people involves citizens contacting the responsible crisis management agencies, such as municipal crisis teams or public health departments, and includes requests for information or specific policy decisions, such as the (early) opening of local stores. Information about the psychosocial situation is also gathered through contact with patients through the health department or via help hotlines for employees in aid organizations	“[We’ve had] thousands and thousands of calls over the months from people looking for advice. They needed tips, they needed help.” (Crisis Management Team Leader/Aid Organization)
Media coverage	Media coverage (e.g., newspapers, television, radio) was less relevant to the creation of a psychosocial situation picture. Occasionally, media reports were evaluated to assess the public perception of one's own organization, to identify strategic fields of action at an early stage (disaster management organization), or to identify regional supply bottlenecks at an early stage (critical infrastructure organization)	“There was relatively little press coverage. And it has had relatively little impact on our picture of the situation.” (Mayor/Municipal Administration)
Social media	Social media platforms, especially Facebook, are used to assess the extent to which public reporting exerts a possible negative influence on the readiness of the civil protection organization’s volunteer members. In the area of municipal crisis management, inquiries, shared misinformation or rumors shared on social media also play a role. Finally, social media channels were used to gauge public opinion on municipal Corona policy in general	“I had my press department with me regularly, and my impression is that they had their ear to the people, at least in the social media […]. And then, what is discussed there, what worries the population, they have just brought into the staff.” (Crisis Management Team Leader/Municipal Administration)
Surveys/studies	Findings based on scientific methods in the form of studies and surveys on the psychosocial situation of the population are hardly taken into account by crisis managers. The participants are often unaware of studies or lack the time and personnel resources to read them	“As I said, in March, April last year I had several weeks where I did 140 h a week. If it was a lot, I slept three hours at night. Because, then you went home, then you are moved by this,[…] and then you researched again at night: How did other municipalities do it? So, to fall back there on any scientific things, no chance.” (Crisis Management Team Leader/Municipal Administration)

4 and

**Table 5:** Summary of the results for RQ 3

Category	Definition/summary	Example
Lower estimation of social science data compared to epidemiological data	The scientific (and political) significance of epidemiological data is considered to be higher than that of social science data. In this context, “hard” epidemiological indicators are considered more meaningful than “soft” social science study data on psychosocial consequences	“But that’s also often difficult, because that’s soft data, as everybody knows that the children are suffering, but the corresponding studies are, yes, the data are somehow softer than these very clear [virus] transmission data. So, you know, one person infects 2.5 others under these conditions. That’s mathematics and you can then calculate something like that and then pretend that that’s the truth.” (State Administration (Ministry of Health)/Crisis Management Team Member)
Priority of infection control	Protection against psychosocial consequences is secondary to the goal of protecting human life. Participants are aware of the psychosocial consequences, but orient their actions primarily to the epidemiological situation or to infection control. Crisis management primarily means protecting people from dying of Covid-19	“Of course, that [psychosocial aspects] plays a major role in all decisions. But […], I’ll put it this way, we don’t sit in a circle of chairs and assess our mental state. So, in crisis management, it’s about saving human lives, and not about examining one’s own or other psychosocial sensitivities.” (Health Department/Chief Medical Officer)
Political and public discourse	The extent to which psychosocial consequences are a relevant argument in the recommendation of policy measures depends on the epidemiological situation as well as on the public discussion of the pandemic situation The health administration crisis staff member points out that a low number of Covid-19 cases during the summer made it possible to take a closer look at studies of psychosocial consequences during the summer of 2020	“It is always at the tipping points, was of course, when such a wave then flattened, there was a great discussion, how to deal with it and then exactly these things became more, they got a higher priority. And now, as I said, since the summer, when people have been saying that vaccination is underway and now we have to slowly see that we slowly get back to normality, as far as that’s somehow possible, this [psychosocial consequences] now really plays a much greater role.” (Crisis Management Team Member/State Administration (Ministry of Health))

5.

## Footnotes

1 This includes psychological and social problems as well as pathological patterns (e.g., post-traumatic stress disorder, anxiety, depression), needs (e.g., shelter, food, information), coping strategies, risks (e.g. socio-economic status, housing situation, previous illnesses) and resources (e.g., knowledge, social networks, financial resources). The term "psychosocial" emphasizes, first, that crises and disasters always affect individuals *and* social communities (Kaniasty and Norris 2004). Second, this term emphasizes that individuals are embedded in social contexts that influence the form and extent to which they are affected by a disaster or a crisis. The risk of being affected, the perception of hazards, or the availability of protective resources also depend on socioeconomic factors (Bonanno et al. 2010; Dückers 2017).

2 For an overview of sensemaking studies, see Brown et al. (2015); Maitlis and Christianson (2014); Maitlis and Sonenshein (2010); Mills et al. (2010); Weick et al. (2005).

3 The entire final category system can be found in the Appendix (Table 2, 3, 4 and 5).

4 Most crisis manager also used documents such as situation reports, recommendations, or legal decrees from federal authorities such as the RKI, Federal Ministry of Health, or Federal Ministry of the Interior and Community as part of their operational crisis management. These sources were primarily used to obtain information on the epidemiological situation as well as the current legal situation, but not to obtain knowledge on the psychosocial consequences. For this reason, these documents were not considered a relevant source of information in the sense of the RQ2 and therefore not included in the category system.
